# ChatGPT Answers the 110-Question Laboratory Enzymology Student Exam: Pass or Fail?

**DOI:** 10.7759/cureus.82168

**Published:** 2025-04-13

**Authors:** Berina Hasanefendic, Aleksandra Pasic, Selvedina Duskan, Emir Sehercehajic, Altaira Jazic Durmisevic

**Affiliations:** 1 Clinical Biochemistry and Laboratory Medicine, Clinical Center University of Sarajevo, Sarajevo, BIH; 2 Department for Laboratory Technologies, Faculty of Health Studies, University of Sarajevo, Sarajevo, BIH; 3 Department for Pathohistology and Cytology, ASA Hospital, Sarajevo, BIH; 4 Emergency Medicine Clinic, Clinical Center University of Sarajevo, Sarajevo, BIH

**Keywords:** artifical intelligence, chatgpt, enzymes, medical education, metabolism

## Abstract

Introduction

Chatbots like ChatGPT have attracted a lot of interest lately due to their ability to generate human-like responses. Their reliability and accuracy are still questionable, and they are the topic of many studies in different fields. Therefore, the aim of this study was to examine the knowledge of two versions of chatbots regarding laboratory enzymology and to compare it with the average knowledge of students for the purpose of considering the use of ChatGPT in providing answers in this field.

Material and methods

An exam with 110 questions covering four topics was answered by students and ChatGPT-3.5 and ChatGPT-4.0. The accuracy of the answers of 52 students and ChatGPT was evaluated. The accuracy of answers between students and artificial intelligence was compared, and the percentage of passing the exam was 60%. All responses were reviewed by two authors with full interrater agreement.

Results

Total scores for students, ChatGPT-3.5, and ChatGPT-4.0 were 85.46%, 52.73%, and 74.55% (p < 0.05), whereby ChatGPT-4.0 achieved better results compared to the other chatbot. ChatGPT-3.5 and ChatGPT-4.0 achieved the best results on questions about enzymes in metabolism. The lowest scores for both chatbots were observed in the laboratory analysis of enzymes.

Conclusion

ChatGPT showed average results in the Laboratory Enzymology exam and scored lower than students. This proved that chatbots could be a potential tool for learning and eventual implementation in higher and/or medical education with extensive optimization but still cannot replace a human.

## Introduction

At the end of 2022, the development of artificial intelligence led to the emergence of ChatGPT based on the Large Language Model (LLM). Chatbots (interactive software applications) based on LLMs have the ability to process text questions and provide text answers based on neural networks and deep learning of millions of data. Generative Pretrained Transformer (GPT) currently provides two versions, GPT-3.5 and GPT-4, which use a generative model to create output text in the form of a human-like answer from an input question [1]. The application of a new form of virtual tutor-ChatGPT-and its application has been investigated in various fields such as economics, industry, natural sciences, medicine, and education. Studies conducted so far show relatively good results and advise the use of these tools in learning and solving many tasks and problems [2,3].

Many studies have been conducted in recent years to analyze the accuracy and reliability of the information provided by ChatGPT in medicine and healthcare [4,5]. Given the demonstrated good results and easy accessibility, many students of medicine and health and related sciences have experienced the opportunities offered. The answers obtained from artificial intelligence are used mainly for writing essays, answering exam questions, and helping or clarifying doubts in acquiring new knowledge and skills. With the aim of implementing artificial intelligence in medical education, many professional associations, the academic community, and individual experts were used as references and credible evaluators of the validity of the level of capabilities of ChatGPT [6,7].

Laboratory enzymology as a subfield of clinical biochemistry represents an important segment in the education of laboratory professionals, focusing on the structure, role, and clinical significance of enzymes in the human body with a focus on laboratory analysis of enzymes. By studying this subfield, graduates acquire expanded knowledge about enzymes and enzymatic reactions, as well as competencies for their clinical and experimental testing [8]. Therefore, the aim of this study is to compare the average student's knowledge base against artificial intelligence on laboratory enzymology, using a student exam to examine the reliability and accuracy of ChatGPT answers. Based on the correct answers, the usefulness of the two versions of ChatGPT in providing accurate information in the field of laboratory enzymology will be evaluated.

## Materials and methods

Study design

The Laboratory Enzymology exam is an integral part of the graduate study of laboratory technology, after which students acquire the knowledge and competencies necessary for medical, clinical, and experimental enzyme testing. The exam consisted of 110 (60 for the first and 50 for the second midterm) multiple-choice questions, where only one answer is correct, or an explanation is required (Appendix). The questions were created by the first author (associate professor). A preliminary list of questions was sent to other co-authors for review, after which grammatical or semantic corrections were suggested, which were accepted. The corrected list of questions represented the final student exam. The following areas were covered: introduction to enzymes and enzymatic reactions, enzymes in metabolism, clinical enzymology, and laboratory analysis of enzymes.

The exam was taken by 52 students in two terms-the first and second midterms, each lasting 45 minutes. To pass the exam, it was necessary to achieve more than 60% correct answers.

ChatGPT

On October 20, 2024, 110 test questions were created in text form for ChatGPT versions 3.5 and 4.0. Each question included instructions, such as "Which of the given answers is correct?" The answers from both versions were recorded and reviewed by two authors. After evaluating the answers, there was no noticeable difference in the test scores between the two versions. Both versions were considered as 53rd and 54th students.

Statistical analysis

After testing for normality of data distribution, non-normal distribution was shown, and non-parametric tests were used for analysis. The Kruskal-Wallis test was used to compare the achieved test scores for three types of participants. All analyses were performed in IBM Corp. Released 2017. IBM SPSS Statistics for Windows, Version 25.0. Armonk, NY: IBM Corp. P<0.05 was considered significant.

## Results

Students and both versions of ChatGPT completed a laboratory enzymology test consisting of 110 questions, mostly from the fields of enzymes and enzyme reactions and clinical enzymology, as shown in Table 1.

**Table 1:** Percentage of subfields in the laboratory enzymology test

Fields of questions	Number of questions (N)	Percentage of questions in the test (%)
Enzymes and enzyme reactions	48	43.64
Enzymes in metabolism	14	12.73
Clinical enzymology	40	36.36
Laboratory analysis of enzymes	8	7.27

Total scores for students, ChatGPT-3.5, and ChatGPT-4.0 are indicators that students and ChatGPT-4.0 had more than 60% correct answers and thus passed the exam, while ChatGPT-3.5 scored lower, as shown in Table 2. When comparing the total scores between students and ChatGPT-3.5, students and ChatGPT-4.0, and ChatGPT-3.5 and ChatGPT-4.0, a significant difference was shown, p = 0.003, p = 0.001, and p = 0.001, respectively. Significant differences were also shown in the differences in scores achieved in the subfields: enzymes and enzymatic reactions-students and ChatGPT-4.0 (p = 0.001); enzymes in metabolism-students and ChatGPT-3.5 (p = 0.046) and students and ChatGPT-4.0 (p = 0.005); clinical enzymology-students and ChatGPT-3.5 (p = 0.002); and laboratory analysis of enzymes-students and ChatGPT-4.0 (p = 0.015).

**Table 2:** Scores achieved on the laboratory enzymology test by students, ChatGPT-3.5, and ChatGPT-4.0 Scores are represented as the number of correct answers, N, and the percentage of correct answers, %, out of the total number of questions. The Kruskal-Wallis test was used to compare the achieved test scores. Statistical significance was set at level p < 0.05.

	Students	GPT-3.5	GPT-4.0	Students vs. GPT-3.5	Students vs. GPT-4.0	GPT-3.5 vs. GPT-4.0
Enzymes and enzyme reactions	40 (85.41)	26 (54.17)	35 (72.91)	0.192	0.001	0.236
Enzymes in metabolism	12 (85.71)	8 (57.14)	11 (78.57)	0.046	0.005	0.269
Clinical enzymology	35 (87.5)	21 (52.5)	31 (77.5)	0.002	0.173	0.323
Laboratory analysis of enzymes	7 (87.5)	3 (37.5)	5 (62.5)	0.618	0.015	0.108
Overall	94 (85.46)	58 (52.73)	82 (74.55)	0.003	0.001	0.001

**Figure 1:**
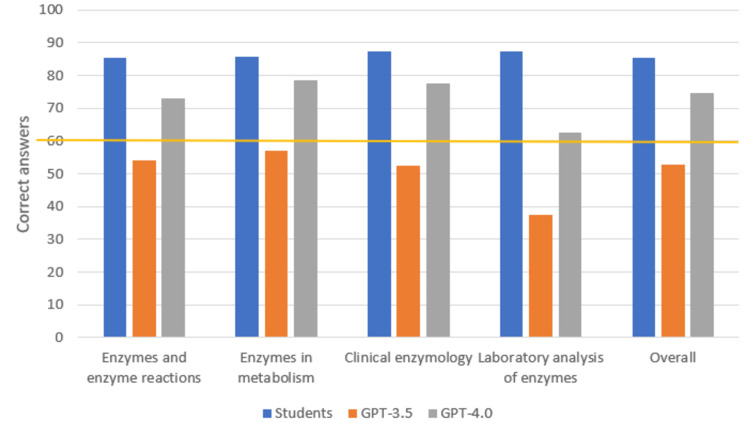
Total scores and subfield scores for students, ChatGPT-3.5, and ChatGPT-4.0

Students achieved the highest score of correct answers in Clinical Enzymology 35/40 (87.5%), ChatGPT-3.5 in Enzymes and Enzyme Reactions 35/48 (54.17%), and ChatGPT-4.0 in Enzymes in Metabolism 11/14 (78.57%), as shown in Figure 1.

## Discussion

In our conducted study, we examined and evaluated the performance of both versions of ChatGPT and students’ knowledge of basic and clinical knowledge in laboratory enzymology. The condition for passing the exam is to obtain more than 60% points, and according to this, students (85.46%) and ChatGPT-4.0 (74.55%) passed, while ChatGPT-3.5 (52.73%) did not achieve a sufficient score to pass the test. These results provide a better and closer insight into the possibility of applying chatbots in medical education-enzymology as part of biochemistry in this case.

Optimizing the learning process and exam preparation undoubtedly keeps pace with the development of new technologies. Newer technologies, such as artificial intelligence, have so far shown their potential and wide use among students due to their easy accessibility via web browsers, even without the necessary creation of a user account. Therefore, researchers from many medical fields have aimed to test the credibility and accuracy of the information obtained, often with reference sources [9]. Previously conducted studies carried out so far have shown high scores on tests of different purposes and different medical fields [10-12].

To the best of our knowledge, previous research did not examine the knowledge of the subdisciplines of medical biochemistry, such as laboratory enzymology. Previous studies have shown that students achieved average scores in medical biochemistry exams: 58% in the study of Surapaneni KM et al. [13], 59.3% in the study of Luke WANV et al. [14], and 70% in the study of Ghosh A et al. [15]. Although ChatGPT proved in the mentioned studies that it can be a useful tool for solving student exams in medical biochemistry, in a study by the same author, Surapaneni KM, several shortcomings (decreasing precision with the increasing complexity of questions, need to validate answers, differences in answers in two independent sessions) and poorer performance in solving clinical cases from this field was shown [16]. Additionally, our study suggests that chatbots can be a useful platform for obtaining information, but with variations between versions in the case of laboratory enzymology.

Limitations

The conducted study has several design and performance limitations. Firstly, the exam for ChatGPT was conducted only once. The accuracy of the answers was not tested more than once. The test questions did not involve recognizing images or solving any mathematical operations, which would be good indicators of the capabilities of artificial intelligence. As a research instrument, a self-administered test prepared by the author based on his knowledge and many years of experience was used instead of a validated instrument. The reason for this is the unavailability of some form of instrument for assessing students' knowledge of laboratory enzymology. At the same time, we believe that our test form could be used as a basis for a validated test form. 

## Conclusions

In our study, we examined and investigated the reliability of ChatGPT in answering questions from different aspects of laboratory enzymology. The results showed that ChatGPT has the ability to answer basic and clinically based-questions in this field. In this study, ChatGPT-4.0 scored better than ChatGPT-3.5.

Furthermore, we conclude that this study can be used to assess the possibility of implementing chatbots in higher and/or medical education. Our results showed that ChatGPT-4.0 is more reliable and accurate and has greater potential in the field of laboratory enzymology compared to ChatGPT-3.5. Based on the average score achieved by both versions, we believe that ChatGPT can be a good learning tool with additional software optimizations, but it still cannot replace a human.

## Appendices

**Table 3:** Laboratory enzymology test with 110 questions and LLM responses

Test subfields	Questions	ChatGPT-3.5	ChatGPT-4.0
Enzymes and enzyme reactions	Which of the following statements is true about enzymes? I They enhance the reaction by stabilizing the transition state. II They increase the activation energy to shift the equilibrium in favor of the reaction product. III They lower the activation energy by changing the products of the reaction.	Incorrect	Incorrect
	Which of the following statements is true about substrate-saturated enzymes? A At the level of substrate saturation, a competitive inhibitor will affect the reaction rate more than a non-competitive inhibitor B An enzyme with a lower Km is easier to saturate than an enzyme with a higher Km C Any excess substrate will increase the equilibrium toward the end product of the reaction.	Incorrect	Incorrect
	Which of the following is true about the induced model? A The active site can be influenced by molecules that bind elsewhere on the enzyme molecule B The initial binding of the enzyme and substrate is the tightest conformational bond C The induced model must occur before the initial binding of the enzyme and substrate for the reaction to proceed	Incorrect	Incorrect
	Which of the following classes of enzymes catalyzes a reaction in which two molecules are covalently bound to each other?	Correct	Correct
	When a piece of liver tissue is placed in hydrogen peroxide, peroxide bubbles form because:	Incorrect	Correct
	The enzyme pepsin works best in which environment?	Correct	Correct
	If an enzyme is found at an inappropriate pH, it?	Correct	Correct
	Which inorganic ion serves as a cofactor for alcohol dehydrogenase?	Incorrect	Correct
	In which body fluid are the highest concentrations of amylase?	Correct	Correct
	Which of the following has very low aspartate transaminase values: heart, liver, saliva?	Correct	Correct
	Which of the following organs has very low aspartate transaminase values: heart, liver, saliva?	Correct	Correct
	Isoenzymes of which enzyme can be separated with electrophoresis?	Incorrect	Correct
	Describe the general principle of determining enzyme activity?	Correct	Correct
	What are the two units for expressing enzymatic activity?	Incorrect	Correct
	Determination of which enzymes in urine is not clinically significant?	Incorrect	Incorrect
	Acid phosphatase enzyme is not normally found in?	Correct	Correct
	Which of the following enzymes (elastase, chymotrypsin, ALP) is not found in feces?	Correct	Correct
	Which cells produce pepsinogen?	Correct	Correct
	Lipase is not normally found in?	Correct	Correct
	An enzyme is normally found in saliva?	Correct	Correct
	Urease is an enzyme that breaks down urea into?	Correct	Correct
	Which enzyme converts lactate into pyruvate?	Correct	Correct
	Do proteases have high specificity?	Incorrect	Incorrect
	Proteases catalyze protein synthesis in cells?	Correct	Correct
	Which of the following enzymes has the lowest relative molecular mass (hydrolase, ribonuclease, LDH)?	Correct	Correct
	Which of the following enzymes has the highest relative molecular mass (hydrolase, peroxidase, ribonuclease)?	Incorrect	Incorrect
	The highest concentrations of lipase are found in which cell organelle?	Correct	Correct
	Glucokinase is an enzyme found in?	Incorrect	Incorrect
	Lipase works best in which pH of the small intestine?	Correct	Correct
	The enzymatic reaction will be accelerated with a higher enzyme concentration?	Incorrect	Incorrect
	To which group of enzymes does the LDH enzyme belong?	Correct	Correct
	What forms holoenzymes?	Incorrect	Correct
	Which enzyme is needed to convert glucose into fructose?	Correct	Correct
	Which of the listed enzymes (ptyalin, proteases, glucose oxidase) is found intracellularly?	Incorrect	Correct
	Which ion is required for optimal enzyme activity of amylase?	Correct	Correct
	Which of the listed enzymes (AST, LDH, ALP) is not found in muscle tissue?	Correct	Correct
	In children, ALP values ​​are much higher than in adults due to which process?	Correct	Correct
	The highest concentrations of which groups of enzymes are found in urine?	Incorrect	Incorrect
	If the urine pH is < 6, what happens with enzyme activity?	Incorrect	Correct
	Which group of enzymes is not normally found in urine?	Incorrect	Incorrect
	The highest concentrations of LDH are found in?	Correct	Correct
	The highest concentrations of AST are found in?	Incorrect	Correct
	AST-1 isoenzyme is also called?	Correct	Correct
	Which enzymes are found in erythrocytes?	Correct	Correct
	AST can be found in which two forms in the cell?	Incorrect	Incorrect
	ALP isoenzymes are?	Incorrect	Correct
	Which enzyme in the CNS play a role in the regulation of vascular tone, local perfusion and neuron function?	Correct	Correct
	At what pH is the optimum of alkaline phosphatase activity?	Incorrect	Correct
	Synthesis of bone-specific alkaline phosphatase (BAP) takes place in?	Incorrect	Incorrect
Enzymes in metabolism	What is the optimal pH for the breakdown of carbohydrates in the mouth?	Correct	Correct
	The key enzyme in the first step of heme metabolism belongs to which enzyme group?	Incorrect	Correct
	Heme catabolism can be represented by a scheme?	Correct	Correct
	What is the starting amino acid for heme synthesis?	Correct	Correct
	In the Krebs citrate cycle, the products are formed in the following order?	Incorrect	Incorrect
	ATP synthesis occurs in?	Correct	Correct
	In which complex does the reduction of FADH2 to FAD occur?	Incorrect	Incorrect
	In the metabolism of amino acids, the transamination process produces?	Incorrect	Correct
	In the process of gluconeogenesis, which enzyme converts glucose-6-phosphate into the final product glucose?	Correct	Correct
	The highest concentrations of enzymes that participate in cellular respiration are found in which organelle?	Correct	Correct
	A very small amount of pepsin (1%) is excreted by the kidneys. In what form?	Correct	Correct
	Which cations are a CK inhibitors?	Correct	Correct
	In the first step of glycolysis, hexokinase produces glucose-6-phosphate. G-6-P can also bind hexokinase to its active site, blocking ATP access. This is an example of?	Incorrect	Incorrect
	In the Krebs citrate cycle, the products are formed in the following order?	Correct	Correct
Clinical enzymology	Which clinical conditions cause changes in amylase concentration?	Correct	Correct
	Lack of homogenisate-1,2 dioxygenase enzyme leads to alkaptonuria due to?	Incorrect	Incorrect
	In a patient with alkaptonuria, the color of urine is?	Correct	Correct
	Which values of pancreatic enzymes are observed in chronic pancreatitis?	Correct	Correct
	The normal value of amylase in serum is?	Correct	Correct
	What changes in enzyme status occur during pregnancy?	Correct	Correct
	Phenylketonuria occurs due to disorders at the level of?	Incorrect	Correct
	G6PD enzyme deficiency can lead to fetal death and spontaneous abortions?	Incorrect	Correct
	Does serine protease play an important role in the control of DM type 2?	Correct	Correct
	Lipase enzyme does not play a significant role in metabolic syndrome?	Correct	Correct
	In pericarditis, the enzyme profile corresponds to which changes in AST and CK?	incorrect	Correct
	During the systematic examination of the patient, an elevated ALP value (280 IU/L) was observed. Which biomarker should be additionally determined in order to differentiate the origin - liver or bone?	Incorrect	Incorrect
	Decreased concentration of pepsinogen in plasma is found in which type of anemia?	Correct	Correct
	De Ritis ratio above 1 does not indicate liver fibrosis?	Correct	Correct
	Which CK isoenzyme is considered a biomarker for brain tumors?	Correct	Correct
	Which enzyme is important to determine when distinguishing between septic and aseptic meningitis?	Incorrect	Correct
	How is activity in tumor cells enzyme involved in proteolysis and aerobic glycolysis?	Incorrect	Correct
	Which LDH isoenzyme has the highest concentration in hemolytic anemia?	Incorrect	Correct
	Which LDH isoenzyme has the highest concentration in cholelithiasis and chronic cholecystitis?	Incorrect	Correct
	Which are AST reference values for men and women?	Correct	Correct
	Does AST value follows ALT in myocardial infarction?	Incorrect	Correct
	What correlates with NSE concentration in acute stroke patients?	Correct	Correct
	The concentration of LDH in the cerebrospinal fluid of adults is less than 40 U/L?	Correct	Correct
	Is NSE included in protein degradation process?	Incorrect	Incorrect
	Which enzyme is found in placenta?	Correct	Correct
	Lipase is produced and released by which organ?	Correct	Correct
	GGT is one of the most sensitive indicators of which disease?	Correct	Correct
	The enzyme specific for liver damage caused by alcohol or drugs is?	Incorrect	Correct
	Mumps can increase which enzyme concentration?	Incorrect	Correct
	Can leukocyte elastase be normally elevated during the pregnancy?	Correct	Correct
	Does LDH have more important significance then ALT in liver diseases?	Incorrect	Correct
	What is AST/ALT ratio in alcoholism?	Correct	Correct
	After how many hours after the onset of myocardial infarction does the CK-MB value begin to rise?	Correct	Correct
	Which enzyme is often used as an indicator of cholestasis?	Incorrect	Correct
	Is concentration of AST usually higher than the concentration of ALT in the serum.	Incorrect	Incorrect
	In which disease is the highest activity of alkaline phosphatase observed?	Incorrect	Correct
	Which enzyme is often used as a marker for the diagnosis of acute myeloid leukemia?	Incorrect	Correct
	Rank the enzymes AST, LDH and CK from the one that decreases first and returns to the reference interval to the one that decreases last and returns to the reference interval after a myocardial infarction?	Incorrect	Incorrect
	Why are LDH concentrations very often elevated in various pathological conditions?	Correct	Correct
	Name two parameters other than enzymes that are important for diagnosing myocardial infarction?	Correct	Correct
Laboratory analysis of enzymes	In everyday practice, a test tube with which anticoagulant is used to determine enzymes?	Correct	Correct
	Which enzyme determination method is most often used in everyday practice?	Correct	Correct
	To determine blood glucose, two methods are used that use two enzymes and those enzymes are?	Incorrect	Correct
	Which of the listed enzymes (amylase, LDH, GGT) is absolutely not recommended for analysis in hemolyzed samples?	Incorrect	Correct
	Does hemolysis affect the analysis of neuron-specific enolase?	Correct	Correct
	Enzymes for laboratory analysis are taken out in a test tube with a (_) cap, and the liquid obtained after centrifugation does not contain (_).	Incorrect	Incorrect
	Enzymes with clinical significance are analyzed in the laboratory on a (_) analyzer, using the (_) method, and their activity is measured in (_) and (_).	Incorrect	Incorrect
	Sample preparation for pancreas elastase analysis includes?	Incorrect	Incorrect
